# Case report: Additional variants induced sudden cardiac death among pediatric ACM with *DSG2* homozygous mutant genotype: a report of three cases

**DOI:** 10.3389/fgene.2024.1428796

**Published:** 2024-08-26

**Authors:** Meng Wei, Yifei Li, Xiaoliang Liu, Kaiyu Zhou, Yu Qiu, Lei Liu, Lili Huang, Zhongqiang Liu

**Affiliations:** Key Laboratory of Birth Defects and Related Diseases of Women and Children of MOE, Department of Pediatrics, West China Second University Hospital, Sichuan University, Chengdu, Sichuan, China

**Keywords:** DSG2, WES, ARVC, additional mutation, sudden cardiac death

## Abstract

**Background:**

Mutations in genes encoding desmosomal proteins are the leading cause of arrhythmogenic cardiomyopathy (ACM). The majority of the inherited ACM cases demonstrate autosomal dominant genotype. Several cases with the homozygous *DSG2* c.1592T>G (p.F531C) variant genotype demonstrate adverse clinical outcomes, but the roles of associated genetic mutations are not clear. In this report, we describe three ACM cases with the homozygous *DSG2* c.1592T>G (p.F531C) variant genotype combined with additional heterozygous cardiomyopathy-related genetic mutations that cause aggravated clinical manifestations and worse clinical outcomes.

**Case presentation:**

The three reported probands demonstrated similar clinical presentations such as heart failure, cardiac enlargement, and lethal arrhythmias. All of them experienced sudden cardiac death (SCD) before undergoing implantable cardioverter defibrillator (ICD) or heart transplantations. Whole-exome sequencing analysis demonstrated that the three patients inherited the homozygous *DSG2* c.1592T>G (p.F531C) variant. Furthermore, probands I, II, and III also inherited additional heterozygous cardiomyopathy-associated mutations, including *DSP* c.7883T>C, *SCN5a* c.3577C>T, or *MYH7* c.427C>T, respectively. These variants were confirmed as pathogenetic variants. A systematic review of all the reported ACM cases with the homozygous *DSG2* variants suggested that the additional genetic mutations contributed to the early age onset of ACM and lethal cardiac events.

**Conclusion:**

In conclusion, we report three rare cases of ACM with the same homozygous *DSG2* variant in combination with additional heterozygous mutations in cardiomyopathy-associated genes. A systematic review of all the ACM cases with homozygous *DSG2* variants demonstrated that the additional genetic variants contributed to the aggravated clinical manifestations and worse clinical symptoms of the ACM patients because of homozygous *DSG2* mutations, including early disease onset and lethal cardiac events. Our data suggested that comprehensive genetic evaluation should be performed to identify any potential additional pathogenic variants that may significantly influence the clinical prognosis and outcomes of patients with ACM. The knowledge of underlying molecular mutations would be useful in designing better therapeutic strategies for ACM patients with multiple genetic mutations.

## 1 Introduction

Arrhythmogenic cardiomyopathy (ACM) is a hereditary cardiac disease that primarily affects the right or bilateral ventricles. It is mainly caused by mutations in genes encoding the desmosomal proteins. The pathological hallmark of ACM includes loss of ventricular cardiomyocytes and their replacement by the fibrous and adipose tissues (Corrado et al., 2017a). ACM is significantly associated with sudden cardiac death (SCD), especially in young adult patients and athletes (Corrado et al., 2017a). te Riele et al. (2015) reported severe ACM phenotype in pediatric patients, including significantly higher risk of SCD and progression towards heart failure; adult patients with ACM were characterized by sustained ventricular tachycardia, which required implantable cardioverter defibrillator (ICD) implantation or heart transplantation (HTx). Therefore, there is an urgent need for novel and effective primary prevention strategies and a hierarchical risk classification system based on the genetic analysis results.

Approximately 50% of clinically diagnosed cases of ACM are caused by mutations in genes encoding the desmosomal proteins (Austin et al., 2019). Molecular dysfunction of the desmosomes, which are critical components of the tight junction between the cardiomyocytes, would cause loss of intercellular adhesion between the cardiomyocytes, detachment, and apoptosis of the cardiomyocytes, and replacement of the cardiomyocytes by the fibrotic and adipose tissue. Exercise exacerbates the adhesion defect, with a greater impact on the thinner walled right ventricle than on the left ventricle (Corrado et al., 2017b). In many cases, ACM is associated with pathogenic genetic variants in the desmosomal genes such as plakoglobin (JUP), plakophilin-2 (PKP2), desmoplakin (DSP), desmoglein-2 (DSG2), and desmocollin-2 (DSC2). Furthermore, mutations in non-desmosomal genes such as PLN-encoding phospholamban and TMEM43-encoding transmembrane protein 43 cause ACM (Fressart et al., 2010; Bhonsale et al., 2015). *PKP2* and *DSG2* are the most common mutant genes involved in the pathogenesis of ACM. Moreover, compared to the *PKP2* mutations, DSG2 variants are more likely to cause heart failure and enlargement of the cardiac chambers. Furthermore, sustainable or non-sustainable ventricular tachycardia is observed in most cases, but SCD is only reported in severe cases. DSG2 is a calcium-dependent adhesion protein of the desmosomes and is synthesized by all the desmosome-bearing tissues (Eshkind et al., 2002).

ACM is a familial disease that shows an autosomal dominant genotype in most cases. The homozygous knock-out mice of desmosomal genes are embryonic lethal. Therefore, homozygous variants of the desmosomal genes are rare. Furthermore, among the reported ACM cases with homozygous *DSG2* variants, the c.1592 T>G (p.F531C) mutation demonstrated severe clinical manifestations, including lethal arrhythmia and heart failure, and required radiofrequency ablation, ICD implantation, and HTx. The p.F531C mutation is located in the extracellular anchor (EA) domain of the DSG2 protein and mediates surface anchoring. Histopathological analysis showed that DSG2 expression was significantly reduced in the *DSG2* p.F531C-positive cardiomyocytes, and was accompanied by the aberrant distribution of Cx43 and the lengthening and twisting of the intercalated disc (Chen et al., 2019a).

In this study, we describe the clinical features and genetic analysis of three pediatric patients with the same homozygous *DSG2* mutation (NM_001943.5: exon11: c.1592T >G; p.F531C) who developed severe heart failure and arrhythmia at a very young age and demonstrated different clinical presentation than the previously reported cases. We investigated whether these three probands were associated with other pathogenetic cardiomyopathy-associated variants that resulted in aggravated clinical presentation and ACM disease progression that finally induced SCD. Furthermore, we performed a systematic review of all the previously reported *DSG2* homozygous variant cases and the complex genetic disorders associated with the probands in this study.

## 2 Methods

This study was approved by the Ethics Committee of the West China Second Hospital, Sichuan University (Approval No. 2021-069). The written informed consent was obtained from the parents of patients before performing whole-exome sequencing (WES) and for including the clinical and imaging details of the patients in the publication.

The genetic tests were performed with samples from all three probands and their parents. The peripheral blood samples were obtained from the patients in an ethylenediaminetetraacetic acid (EDTA) anticoagulant blood sample tube and were stored at 4°C for less than 6 h. DNA was extracted using the Blood Genome Column Medium Extraction Kit (Tiangen Biotech, Beijing, China) according to the manufacturer’s instructions. WES was performed using the NovaSeq 6000 platform (Illumina, San Diego, CA, United States ). The raw sequencing data was processed using FastP to remove the adapters and filter the low-quality reads. Paired-end reads were aligned with the Ensembl GRCh38/hg38 reference genome using the Burrows-Wheeler Aligner. Variant annotation was performed in accordance with the database-sourced minor allele frequencies (MAFs) and the practical guidelines of pathogenicity according to the American College of Medical Genetics. The annotation of MAFs was performed using the 1,000 Genomes, dbSNP, ESP, ExAC, and Chigene inhouse MAF database, Provean, Sift, Polypen2_hdiv, and Polypen2_hvar databases with the R software (R Foundation for Statistical Computing, Vienna, Austria).

## 3 Case presentation

### 3.1 Clinical presentation and laboratory examination

#### 3.1.1 Case 1

**FIGURE 1:**
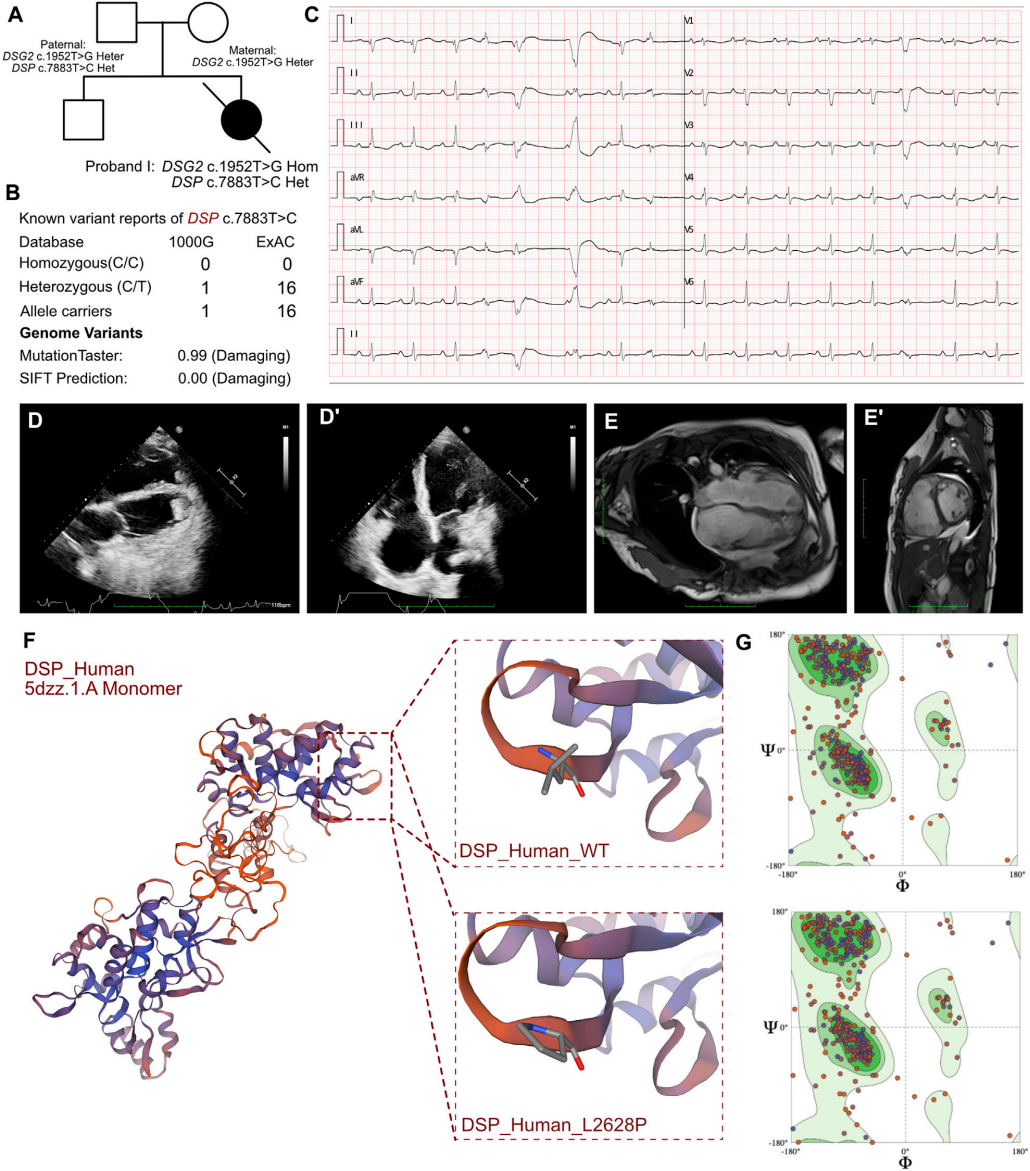
Clinical manifestation of the proband I and molecular features of *DSP*. **(A)**. The proband exhibited a homozygous variant of *DSG2* (c.1592T>G p.F531C) and a heterozygous variant of *DSP* (c.7883T>C p.L2628P). **(B)** The variant of *DSP* c.7883T>C had been reported in 1000G and ExAC, it has predicted protein damaging by MutationTaster. **(C)** ECG of the proband. **(D, D’)** Echocardiography demonstrated thrombus in apex of ventricles. **(E, E’)** CMR demonstrated cardiac enlargement and delayed enhancement. **(F)** The protein structure of DSP has been built based on the 5dzz.1.A template. **(G)** Ramachandran plots indicated that amino acid positions were altered.

Proband I, a 9-year-old female, was admitted to the hospital with a history of decreased activity tolerance over the past year, vomiting for the past 2 weeks, fatigue for the past 3 days, and edema of the extremities for 1 day (Figure 1A). The patient developed dyspnea after exercise and cardiac fatigue a year ago but the patient remained untreated. The patient vomited 2 weeks prior to admission at our hospital. She was taken to a local hospital, where the doctors considered potential heart failure and shock. She showed slight improvement after receiving oxygen, cardiac tonics, diuretics, and other treatments, and was referred to our hospital for further management.

At admission, the heart rate of the patient was 129 beats per minute. The blood pressure measurements were as follows: 101/86 mmHg in the left upper extremity; 103/68 mmHg in the right upper extremity; 97/75 mmHg in the left lower extremity; and 100/74 mmHg in the right lower extremity. This raised suspicion of venous thrombosis in the upper extremities. The respiratory rate was 20 breaths per minute. Physical examination demonstrated pitting edema in both the lower extremities, moderate jaundice of the skin, yellowing of the conjunctiva, pharyngeal congestion, absence of tonsillar enlargement or abnormal secretions, rough respiratory sounds in both the lungs without dry or wet rales, weak and irregular heart sounds without murmurs, a soft abdomen without pressure or rebound pain, a palpable liver 3 cm below the rib cage with a dull central edge, no tenderness or hardness, and a normal-sized spleen. Neurological examination did not show any abnormalities. The extremities were warm with a capillary refill time of 3 s.

The initial blood gas analysis showed a pH of 7.572. The minimum potassium level was 2.6 mmol/L. The elevated levels of B-type natriuretic peptide (8,602.00 pg/mL; n.v. < 100 pg/mL) and troponin I (0.219 μg/L, n.v. < 0.06 μg/L) indicated significant myocardial injury. An immediate electrocardiogram demonstrated first-degree atrioventricular block with T-wave flattening or inversion in leads II, III, aVF, and V3-V6. Holter monitoring findings included atrial tachycardia, sinus arrhythmia, premature atrial contractions with differential intraventricular conduction, polymorphic ventricular premature contractions with visible ventricular fusion waves, QS pattern in leads I and AVL, and flattened or inverted T-waves in leads II, III, aVF, and V4-V6 (Figure 1C). These observations indicated heart failure and arrhythmia. Nucleic acid tests were conducted to identify respiratory pathogens and were positive for the influenza A virus. The tests for other respiratory pathogens were negative. Echocardiography revealed cardiac enlargement, suspected left ventricular thrombus, tricuspid valve closure insufficiency, severe tricuspid regurgitation, and reduced left ventricular ejection fraction (LVEF, 32%) (Figures 1D, D’). The upper extremity venous ultrasound indicated thrombosis of the right cephalic vein at the forearm segment near the wrist, and thrombosis of the entire left cephalic vein and the upper arm segment. Cardiac magnetic resonance (CMR) imaging demonstrated overall cardiac enlargement (LVEF = 21%, LA = 28 mm, LV = 56.3 mm, RA = 57 mm, RV = 50.2 mm), especially enlarged right heart with mitral and tricuspid regurgitation. Delayed enhancement imaging showed scattered strips of delayed enhancement foci in the myocardium of both ventricles with localized transmissibility (Figures 1E, E’). This indicated primary cardiomyopathy. The presence of multiple nodular shadows in both the ventricles was indicative of thrombi, but definitive signs of myocardial edema were not observed. The cardiac functional analysis was hindered by arrhythmia. Therefore, combined assessment with echocardiography was necessary.

#### 3.1.2 Case 2

**FIGURE 2:**
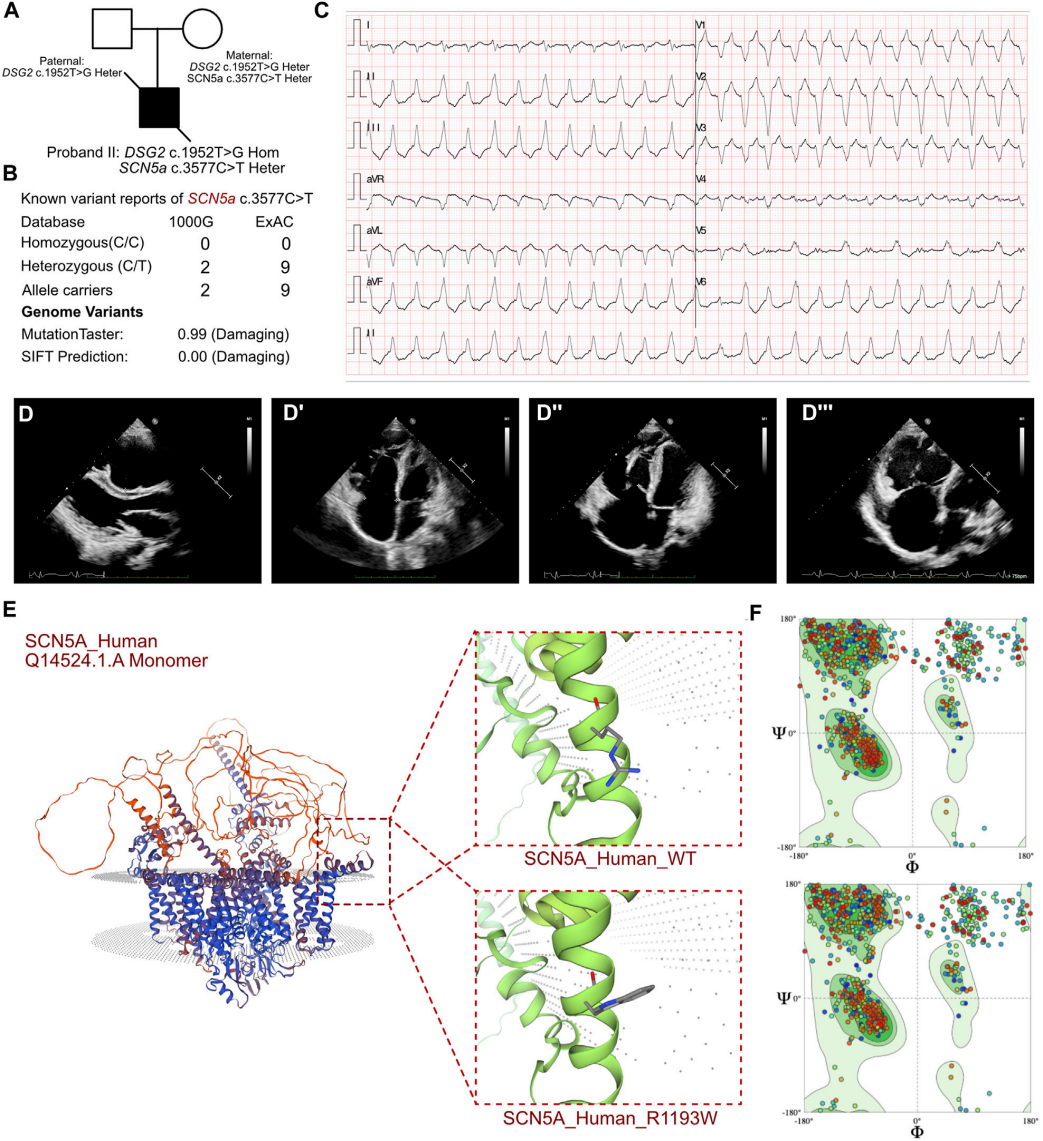
Clinical manifestation of the proband II and molecular features of *SCN5a*. **(A)** The proband exhibited a homozygous variant of *DSG2* (c.1592T>G p.F531C) and a heterozygous variant of *SCN5a* c.3577C>T (p.R1193W). **(B)** The variant of *SCN5a* c.3577C>T had been reported in 1000G and ExAC, it has predicted protein damaging by MutationTaster. **(C)** ECG of the proband. **(D, D’)** Echocardiography demonstrated thrombus in right ventricle and cardiac enlargement, marked dilation of the right atrium and right ventricle, along with inhomogeneous thinning of the ventricular wall with delayed enhancement in the left and right ventricular wall and septum. **(E)** The protein structure of SCN5a has been built based on the Q14524.1.A template. **(F)** Ramachandran plots indicated that amino acid positions were altered.

Proband II, an 11-year-old male, was admitted to the hospital due to heart failure and severe sustainable ventricular tachycardia, which was persistent for 9 days (Figure 2A). Previously, the patient was treated in other medical facilities. He underwent electrical cardioversion and received maintenance amiodarone, diuretics, and anti-infective therapy. Subsequently, he was referred to our department for further management.

During the initial emergency examination, the heart rate was 71 beats per minute, and the blood pressure was 115/86 mmHg. His facial expression was normal without any abnormalities in the skin or the mucous membranes. Pharyngeal congestion was observed without purulent discharge. He exhibited hepatojugular venous reflux. The dilated jugular veins exhibited significant pulsations. Dull heart sounds were detected upon auscultation. The heart boundaries were enlarged and arrhythmia was detected. However, vascular murmurs were not detected in the valvular regions. The breath sounds in both lungs were slightly coarse. The abdomen was soft. The liver and the spleen were not palpable below the ribs. Neurological examination did not show any specific abnormalities. The capillary refill time was 3 s.

The routine blood tests and the blood gas analysis were normal. The blood cultures were negative for six respiratory viruses. The antinuclear antibody profile did not show any significant abnormalities. However, elevated levels of troponin (0.242 ng/mL; n.v. < 0.06 ug/L), myoglobin (113.4 ng/mL; n.v. < 110 ug/L), and B-type natriuretic peptide (BNP) (10,409.0 pg/mL; n.v. < 100 pg/mL) indicated significant myocardial damage. Electrocardiographic findings included sinus bradycardia, accelerated ventricular autonomic rhythm, frequent ventricular premature contractions, intermittent and incomplete right bundle branch block, prolonged QT interval, and atrial premature beats. ECG results also demonstrated sustainable ventricular tachycardia (Figure 2C). 24 h Holter showed sinus arrhythmia, paroxysmal ventricular arrhythmia, atrial premature beats, ventricular premature beats, epsilon waves visible in leads V1-V3, prolongation of QT interval, and flattened or inverted T-waves in leads II, III, AVF, V3-V5. Echocardiography demonstrated an enlarged right heart that was suggestive of cardiomyopathy, suspected thrombus formation in the right ventricle, and heavy tricuspid valve regurgitation. The ventricular wall movement was uncoordinated and the right ventricular systolic function was diminished, as evidenced by a reduction of LVEF to 22% and a low tricuspid annular plane systolic excursion (TAPSE) score of 10 mm (Figures 2D, D’). CMR data demonstrated significant dilation of the right atrium and the right ventricle (LVEF = 35%, LA = 24 mm, LV = 41 mm, RA = 51 mm, RV = 41 mm), and non-homogeneous thinning of the ventricular wall. Mixed delayed enhancement in the left and right ventricular wall and septum indicated reverse septal motion. Diminished right ventricular wall phasic activity and limited diastolic distension indicated ventricular aneurysm. These findings were consistent with a diagnosis of arrhythmogenic right ventricular cardiomyopathy.

#### 3.1.3 Case 3

**FIGURE 3:**
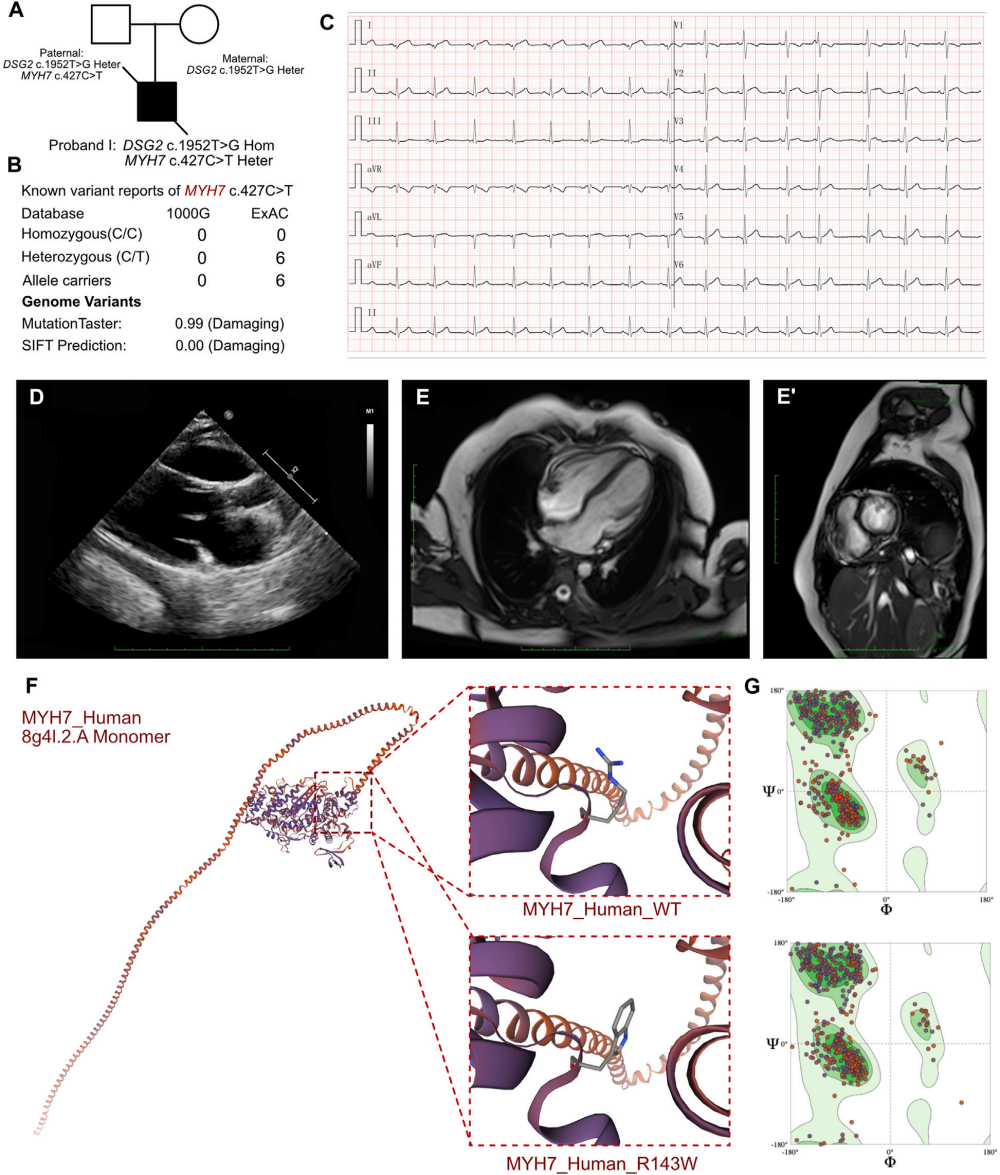
Clinical manifestation of the proband III and molecular features of *MYH7*. **(A)** The proband exhibited a homozygous variant of *DSG2* (c.1592T>G p.F531C) and a heterozygous variant of *NYH7* c.427C>T (p.R143W). **(B)** The variant of *MYH7* c.427C>T had been reported in 1000G and ExAC, it has predicted protein damaging by MutationTaster. **(C)** ECG of the proband. **(D)** Echocardiography demonstrated enlarged left ventricle. **(E, E’)** CMR demonstrated demonstrated patchy abnormal signals in the apical left ventricular free wall, indicative of myocardial edema. **(F)** The protein structure of MYH7 has been built based on the 8g4l.1.A template. **(G)** Ramachandran plots indicated that amino acid positions were altered.

Proband III, a 6-year-old male, was admitted to the hospital with persistent abdominal pain for 5 days and chest pain for 2 days (Figure 3A). During episodes of chest pain, he reported palpitations and a slight decrease in activity tolerance but denied experiencing syncope or shortness of breath. This was not associated with symptoms of fever, cough, or dyspepsia. During the initial emergency examination, the patient presented signs of acute critical illness, including severe sweating, dizziness, and malaise, with pallor and irritability. His heart rate was 24 beats per minute and the blood pressure was 91/52 mmHg. Respiratory sounds were rough bilaterally and dry or wet rales were not detected upon auscultation. Cardiac arrhythmia was detected with subdued heart sounds but pathologic murmurs were absent. The abdominal and neurological examinations did not show any significant findings.

The routine blood cell tests, blood gas analysis, and liver and renal function tests yielded normal results. However, elevated serum levels of cardiac troponin I (2.808 μg/L; n.v. < 0.06 μg/L) and B-type natriuretic peptide (1,020.24 pg/mL; n.v. < 100 pg/mL) indicated significant myocardial injury. An immediate electrocardiogram demonstrated abnormal Q waves in leads I, II, aVF, V4, V5, and V6, and rightward deviation of the cardiac axis (Figure 3C). Holter monitoring detected sinus arrhythmia, premature atrial contractions, and premature ventricular contractions. Based on the clinical presentation, we initially suspected the presence of acute coronary syndrome or viral myocarditis. However, antibody tests for the viral pathogens potentially associated with myocarditis, including cytomegalovirus, Epstein-Barr virus, adenovirus, coxsackievirus, herpes simplex viruses, and human parvovirus B19, yielded negative results. Furthermore, autoimmune antibodies and rheumatologic tests were negative. Echocardiography demonstrated mild enlargement of the left ventricle (42 mm) and a slight reduction in LVEF to 36% upon admission (Figure 3D). CMR demonstrated patchy abnormal signals in the apical left ventricular free wall, which indicated myocardial edema (Figures 3E, E’). No significant abnormalities were detected in the left ventricular structure, with normal systolic function. Delayed enhancement was noted in multiple regions of the left ventricular free wall and interventricular septum, suggesting myocardial fibrosis (LVEF = 40.53%, LV = 42.7 mm, LA = 37.5 mm, RV = 42.1 mm, RA = 48.4 mm).

### 3.2 Molecular results

Molecular analysis showed that all three patients shared a homozygous missense *DSG2* gene variant (NM_001943.5: exon11: c.1592T>G; p.F531C). This homozygous *DSG2* gene variant site has previously been reported in several cases and is associated with the pathogenicity of ACM and heart failure. Moreover, the homozygous variant of DSG2 (c.1592T>G) is significantly associated with lethal arrhythmia and sudden cardiac death (SCD). ICD implantation is highly recommended for such patients. We confirmed that all the parents of these 3 patients were allele carriers (DSG2, c.1592T>G). There was no previous documentation of ventricular tachycardia, SCD, and severe heart failure for any of the parents of these 3 patients. But the father of proband I, mother of proband II, and father of proband III presented with an enlarged right or left ventricle with mild reduced LVEF (45%–50%). The SWISS-MODEL tool was used to analyze the protein stability based on amino acid changes in the *DSG2* variant (c.1592T>G). Ramachandran plots demonstrated that the amino acid positions were altered. The molecular structure of the variant protein based on the AF-Q14126-F1 template resulted in the residue changes between Phe and Cys (Harrison et al., 2016). Therefore, the mutation in the DNA sequence of *DSG2* altered the amino acid sequence, which affected the protein structure and altered the splice site. MutationTaster analysis showed a high probability of this variant being a pathogenic mutation with a score of 0.99. Polyphen2 analysis demonstrated that this mutation was damaging with a score of 1.00 for the mutant protein. Meanwhile, this variant was also listed as a conflicting interpretation of pathogenicity in ARVC in ClinVar database.

WES results from the three probands identified additional cardiomyopathy-related variants. In proband I, we confirmed a heterozygous variant of *DSP* c.7883T>C (p.L2628P), which was paternally inherited (Figure 1A). And its AMCG classification was PM1+PP3. MutationTaster analysis showed that the *DSP* c.7883T>C (p.L2628P) variant was a pathogenic mutation with a probability value of 0.99. SIFT analysis predicted that the mutational change in the protein was damaging (SIFT score: 0.00). Several allele carriers were retrieved from the 1000G (1 heterozygous record) and ExAC (16 heterozygous records) databases (Figure 1B). This variant was also listed as likely benign in 1 “cardiovascular phenotype”\“Arrhythmogenic cardiomyopathy with wooly hair and keratoderma ARVD 8” in ClinVar database. Moreover, the pathogenic effects of this mutation on the protein structure were validated using a crystal structure based on the 5dzz.1.A template between the wild-type and mutant DSP (p.L2628P) amino acid sequences (Figure 1F). Ramachandran plots demonstrated that the amino acid positions were altered in the mutant (Figure 1G).

In proband II, we confirmed a heterozygous variant of SCN5a c.3577C>T (p.R1193W), which was maternally inherited (Figure 2A). And its AMCG classification was PM1+PP3. MutationTaster analysis showed that this variant was a pathogenic mutation with a probability value of 0.99. SIFT analysis showed that the mutation caused a damaging change in the protein (SIFT score: 0.00). Several allele carriers were retrieved from the 1000G (2 heterozygous records) and ExAC (9 heterozygous records) databases (Figure 2B). The pathogenic effects of this mutation on the protein structure were validated using a crystal structure based on the Q14524.1.A template between the wild-type and mutant (p.R1193W) amino acid sequences using AlphaFold2 (Figure 2E). Ramachandran plots demonstrated that the amino acid positions in the SCN5a (p.R1193W) mutant were altered (Figure 2F).

In proband III, we confirmed a heterozygous variant of MYH7 c.427C>T (p.R143W), which was maternally inherited (Figure 3A). And its AMCG classification was PS4+PM1+PP3. MutationTaster analysis demonstrated that this mutation was a pathogenic mutation with a probability value of 0.99. SIFT analysis showed that the mutation caused a damaging change in the mutant MYH7protein (SIFT score: 0.00). Several allele carriers were retrieved from the ExAC database (6 heterozygous records) (Figure 3B). Meanwhile, this variant was also listed as pathogenic/likely pathogenic in hypertrophic cardiomyopathy in ClinVar database. Furthermore, the pathogenic effect of this mutation on the MYH7 (p.R143W) protein structure was confirmed by using a crystal structure based on the 8g4l.2.A template between wild-type and mutant (p.R143W) amino acid sequences with AlphaFold2 (Figure 3F). Ramachandran plots demonstrated that the amino acid positions were altered in the MYH7 (p.R143W) protein (Figure 3G).

Furthermore, we excluded all the other potential variants associated with cardiovascular diseases, arrhythmia, ion channel associated disease, and muscle disorders in the two probands. Moreover, we analyzed for the presence of any chromosomal abnormalities and large-scale deletions. Besides, we also analyzed and observed that all the other variants reported as pathogenic or likely pathogenic were not associated with the phenotype of this proband. Based on the molecular testing analyses, all three probands shared the same *DSG2* homozygous variant in combination with a different pathogenetic heterozygous variant associated with cardiomyopathy. Such patients demonstrated a more complicated genetic disorder, including earlier age of onset cardiomyopathy, lethal arrhythmia, and heart failure, all of which resulted in a higher SCD risk.

### 3.3 Final diagnosis and clinical outcomes

Patients with ARVC were diagnosed based on the International Task Force Criteria of ARVC in 2010 (Table 1) (Marcus et al., 2010). The three patients were provided with invasive or non-invasive mechanical ventilation, antibiotics, cardioprotection, diuretics, milrinone, empagliflozin, Entresto, and metoprolol. In probands I and II, amiodarone was used to manage arrhythmia, especially sustainable or non-sustainable ventricular tachycardia. Due to the complicated genetic disorders, ICD implantation was highly recommended for all three patients. The parents of proband I agreed for the ICD implantation but the parents of probands II and III refused the suggestion. Therefore, probands II and III were included in the waiting list for receiving heart transplantation (HTx).

**TABLE 1:** Clinical diagnosis of three probands.

	Tissue characterization		Repolarization abnormalities		Depolarization/conduction abnormalities		Arrhythmias		Family history		Ventricular alterations		Diagnosis	
	Major	Minor	Major	Minor	Major	Minor	Major	Minor	Major	Minor	Major	Minor	Major	Minor
Proband I	NA^a^		1				1		1				3	
Proband II	NA		1				1		1				3	
Proband III	NA				1		1		1				3	

^a^ Pathological examination was not performed in three patients.

However, proband I died because of SCD at the hospital before the scheduled ICD implantation despite receiving intensive treatment immediately. Proband II was discharged after 1-month of medical treatment at the hospital. His heart function improved after continuous administration of medications, including diuretics, milrinone, empagliflozin, and Entresto. He still suffered from ventricular arrhythmia attacks and palpitations and died because of SCD at 1.5 years after initial diagnosis. In proband III, the heart function recovered after 20 days of treatment, but non-sustainable ventricular tachycardia was recorded. Proband III reported two episodes of syncope during the first year since initial diagnosis. His parents still refused to receive an ICD implantation and preferred HTx but the 8-year-old boy died because of SCD.

### 3.4 Systematic review of all the reported cases of ACM carrying *DSG2* homozygous variants

To investigate the clinical characteristics associated with the homozygous DSG2 variants, and further analyze the differential prognosis among patients with DSG2 variants, we systematically reviewed published articles regarding all the reported cases. Systematic review of the published articles was performed in the Pubmed database using the following search strategy: “DSG2” [All Fields] AND (“cardiomyopathie” [All Fields] OR “cardiomyopathies” [MeSH Terms] OR “cardiomyopathies” [All Fields] OR “cardiomyopathy” [All Fields] OR ((“arrhythmia s” [All Fields] OR “arrhythmias, cardiac” [MeSH Terms] OR (“arrhythmias” [All Fields] AND “cardiac” [All Fields]) OR “cardiac arrhythmias” [All Fields] OR “arrhythmia” [All Fields] OR “arrhythmias” [All Fields]) AND (“heart failure” [MeSH Terms] OR (“heart” [All Fields] AND “failure” [All Fields]) OR “heart failure” [All Fields]) AND (“death, sudden, cardiac” [MeSH Terms] OR (“death” [All Fields] AND “sudden” [All Fields] AND “cardiac” [All Fields]) OR “cardiac sudden death"[All Fields] OR (“sudden” [All Fields] AND “cardiac” [All Fields] AND “death” [All Fields]) OR “sudden cardiac death” [All Fields]))).” We identified 193 articles. Two reviewers (Yifei Li and Meng Wei) reviewed all the titles and abstracts initially and then performed full-text reviews of all the potential articles. Finally, we selected 7 articles that reported 16 cases regarding the homozygous *DSG2* variant (Table 2). This included 12 patients (age range: 14–51 years) with the DSG2 c.1592T>G variant. The most common ECG characteristics were T wave inversion and sustainable or non-sustainable ventricular tachycardia. Patients with the DSG2 c.1592T>G variant were provided with ICD implantation, HTx, or ablation therapies. The remaining 4 cases (age range: 18–56 years) were diagnosed with three homozygous *DSG2* variants, including c.209G>A (1 case), c.874C>T (1 case), and c.1003A>G (2 cases). The patient with the *DSG2* c.209G>A variant received HTx because of severe heart failure at the age of 44 years. The remaining two cases with the *DSG2* c.1003A>G variant received ICD implantation at the age of 52 years and 56 years, respectively.

**TABLE 2:** Clinical information of the reported patients with *DSG2* homozygous variant.

Id	Variant	Clin var Id	Allele	MAF*	Gender	Age of onset	Therapy	Symptom	Electrophysiology	MACE^#^	NYHA
This report											
1	*DSG2* c.1592T>G *DSP* c.7883T>C	654,498 357,965	Homo Heter	1.00E-04 2.00E-04	Female	9	—	Syncope, collapse	TWI, NSVT, VE, AT	1	I
2	*DSG2* c.1592T>G *SCN5a* c.3577C>T	654,498 —	Homo Heter	1.00E-04 6.00E-04	Male	11	—	Syncope, collapse	TWI, Epsilon, sVT	1	II
3	*DSG2* c.1592T>G *MYH7* c.427C>T	654,498 164,401	Homo Heter	1.00E-04 5.00E-05	Male	6	—	Syncope, collapse	AE, DQW	0	II
Chen et al. (2019a)											
1	*DSG2* c.1592T>G	654,498	Homo	1.00E-04	Male	14	HTx	Palpitation, dyspnea	TWI, NSVT	0	III
2	*DSG2* c.1592T>G	654,498	Homo	1.00E-04	Male	29	ICD, RFA	Palpitation, dyspnea	TWI, sVT	1	I
3	*DSG2* c.1592T>G	654,498	Homo	1.00E-04	Female	34	—	Palpitation	TWI, sVT, VE	1	I
4	*DSG2* c.1592T>G	654,498	Homo	1.00E-04	Male	27	ICD	Fatigue	TWI, NSVT, VE	0	III
5	*DSG2* c.1592T>G	654,498	Homo	1.00E-04	Male	18	—	Syncope	TWI, Epsilon, sVT, VE	1	I
6	*DSG2* c.1592T>G	654,498	Homo	1.00E-04	Male	26	—	0	TWI, sVT, VE	1	I
7	*DSG2* c.1592T>G	654,498	Homo	1.00E-04	Female	25	ICD, RFA	Palpitation, dyspnea	TWI, sVT, VE	1	I
8	*DSG2* c.1592T>G	654,498	Homo	1.00E-04	Female	23	HTx	Palpitation, syncope, dyspnea	TWI, sVT, VE, AF	1	IV
Lin et al. (2017)											
1	*DSG2* c.1592T>G	654,498	Homo	1.00E-04	Male	51	RFA	HF	TWI, Epsilon, sVT, VE	1	II
2	*DSG2* c.1592T>G	654,498	Homo	1.00E-04	Male	45	—	HF	TWI, Epsilon, sVT, VE	1	II
Yu et al. (2008)											
1	*DSG2* c.1592T>G	654,498	Homo	1.00E-04	Male	50	ICD	Dyspnea, chest tightness	TWI, sVT	0	III
Chen et al. (2019b)											
1	*DSG2* c.1592T>G	654,498	Homo	1.00E-04	Male	29	RAF	Palpitation, chest tightness	TWI, sVT	1	II
Posch et al. (2008)											
1	*DSG2* c.209G>A	—	Homo	—	Male	44	HTx	Dyspnea	LBBB, AVBI	0	IV
Sato et al. (2011)											
1	*DSG2* c.874C>T	—	Homo	—	Male	18	—	Syncope	—	0	I
Qadri et al. (2017)											
1	*DSG2* c.1003A>G	44,278	Homo	—	Male	52	ICD	Palpitation	sVT	1	II
2	*DSG2* c.1003A>G	44,278	Homo	—	Male	56	ICD	Chest pain	Epsilon, sVT	1	II

MACE (Major Adverse Cardiac Events) contained history of sustained ventricular tachycardia, ICD appropriate therapy and syncope.

HTx, heart transplantation; ICD, implantable cardioverter defibrillator; RFA, radiofrequency ablation; TWI, T wave inversion; sVT, sustained ventricular tachycardia; NSVT, non-sustained ventricular tachycardia; AF, atrial fibrillation; AE, atrial extrasystole; VE, ventricular extrasystole; DQW, Deepening Q wave; Homo, homozygous; Heter, heterozygous.

Compared with the three cases reported in this study, all the other patients did not show any other cardiomyopathy- or arrhythmia-associated genetic variants. Moreover, the age of disease onset was at a later stage. Furthermore, syncope was rarely reported among patients with the individual homozygous DSG2 variant. This suggested that the presence of an additional variant gene significantly increased the risk of pediatric onset ACM and severe adverse clinical outcomes. Therefore, emergency ICD implantation should be recommended for such patients.

## 4 Discussion

ACM is significantly associated with sudden cardiac death (SCD) syndrome among young adults. A higher number of ACM cases are being detected during childhood because of advances in imaging techniques and genetic sequencing. However, there is a higher possibility of misdiagnosing ACM. Therefore, its prevalence may be underestimated because of diverse and atypical clinical presentations, including symptoms like palpitations, syncope, chest pain, dyspnea, and SCD. Typically, ACM is manifested between 20 and 50 years of age, with an average onset age of 30 years (Groeneweg et al., 2015; Nava et al., 2000; Hulot et al., 2004). About 40% of diagnosed ACM cases are asymptomatic and are often discovered during familial screening because of the identification of ACM or other inherited cardiovascular diseases in a family member (Corrado et al., 2017a; Chen et al., 2019a). Hence, genetic testing is of paramount importance in elucidating the underlying cause of the disease, especially in cases of unexplained heart failure, severe arrhythmias, and sudden cardiac arrest among the young population.

Patients with ACM typically present with ventricular arrhythmias, often accompanied by heart failure (Gilotra et al., 2017; McCullough and Perera, 2022). The pathological hallmark of classical ACM predominantly involves significant right ventricular disease, although left ventricular involvement is also observed in a few cases (Sen-Chowdhry et al., 2007). Heart failure at the time of initial diagnosis is uncommon, and its development usually indicates disease progression (Gilotra et al., 2017; Kimura et al., 2017; Cardona-Guarache et al., 2019). ACM is caused by mutations in various genes, especially genes encoding the desmosomal proteins. Familial inheritance accounts for over 30% of the ACM cases and typically follows the autosomal dominant pattern. However, rare cases of autosomal recessive inheritance have also been reported, as reported in patients with the Naxos disease and the Carvajal syndrome. These cases are often presented as part of a cardiocutaneous syndrome and are characterized by hyperkeratosis of the palms and soles and woolly hair (Dalal et al., 2005; Basso et al., 2008; James et al., 2020; McCullough and Perera, 2022; Smedsrud et al., 2022). Moreover, sudden cardiac arrest is an early manifestation of ACM and precedes heart failure and sustained ventricular tachycardia. Among 577 patients in the United States and the Netherlands, 6% experienced sudden cardiac death. An Italian study found that 20% of deaths among young people and athletes were caused by previously undiagnosed ARVC. In a nationwide multicenter observational cohort in the Netherlands, of 12 pediatric-onset ARVC patients and 68 pediatric relatives, 3 (25%) primary patients and 3 (4%) relatives first presented with sudden cardiac death/arrest (Bhonsale et al., 2015; Tennyson and Yapejian, 2022). The genotype of ACM significantly influences the clinical outcomes with compound heterozygous and rare homozygous variants associated with worse prognosis and increased risk of adverse events (Cox et al., 2011; Kapplinger et al., 2011; Bao et al., 2013). Patients with multiple gene variants often experience earlier onset of ACM, more frequent ventricular tachyarrhythmias, and irreversible heart failure (Bhonsale et al., 2015). Atypical clinical presentations of ACM termed as the “hot phase” are also reported, especially in pediatric patients with the *DSG2* gene variants and adults with the *PKP2* variants (Liu et al., 2023). The *DSG2* gene is located on chromosome 18q12.1 and encodes the desmoglein-2 protein. *DSG2* variants such as p.F531C result in altered protein function and cause destabilization of the cardiomyocyte structure, atrophy, and fibrofatty infiltration. The homozygous variant of *DSG2* c.1592T>G (p.F531C) is manifested as a definite ACM phenotype, whereas the heterozygous carriers may remain asymptomatic or present with mild symptoms (Lin et al., 2017). The DSG2 p. F531C variant has been reported in previous publications (Bao et al., 2013; Ng et al., 2013). Furthermore, the homozygous variant of *DSG2* c.1592T>G (p.F531C) has also been identified among several cases and families. The homozygous *DSG2* c.1592T>G variant carriers exhibited a definite ACM phenotype, whereas the heterozygous variant carriers were unaffected or presented only mild ACM-related symptoms in 25% of their relatives (Lin et al., 2017; Chen et al., 2019a).

In the last decade, several studies have investigated the underlying molecular mechanisms in ACM and the clinical associations between the genotype and the phenotype. The current results have suggested that compound heterozygous variants and joint heterozygous variants in two or more cardiomyopathy-related genes are associated with early-onset inherited cardiovascular diseases. Furthermore, patients with more than one ACM- or cardiomyopathy-associated variants demonstrate significantly worse clinical course, earlier onset of symptoms, sustained arrhythmia, lethal ventricular tachycardia, or even sustained ventricular tachycardia. Moreover, such patients demonstrated a 5-fold higher risk of developing left ventricular insufficiency and heart failure than those who carried only a single ACM-related gene variant (Bhonsale et al., 2015). Our study confirmed additional genetic variations in the *DSP*, *SCN5a*, or the *MYH7* genes in the three probands in addition to *DSG2* c.1592T>G (p.F531C), which was the dominant disease causing homozygous variant. The *SCN5A* gene encodes the alpha subunit of the cardiac sodium channel Na_v1.5, which is essential for the proper functioning of the heart’s electrical activity. Mutations in the *SCN5A* gene can disrupt the normal function of the Na_v1.5 channel, leading to arrhythmias and other cardiac issues. As seen in a large pediatric cohort of *SCN5A* variant subjects, cardiac conduction disorders were the most prevalent phenotype (Baruteau et al., 2018). The *DSP* gene encodes the protein DSP, which is critical for the integrity of desmosomes—structures that provide mechanical strength to tissues by binding cells together. The *MYH7* gene encodes the beta heavy chain of cardiac myosin, which is a major component of the contractile apparatus in the heart muscle. Variants in the *MYH7* gene are associated with various types of cardiomyopathy, including hypertrophic cardiomyopathy (HCM), dilated cardiomyopathy (DCM), and restrictive cardiomyopathy (RCM). Both DSP and MYH7 are contractile feature proteins, and their variants play significant roles in the development of cardiac diseases. In our study, the three probands all have compound mutations involving a homozygous DSG2 variant combined with heterozygous mutations in these three genes. As a result, they exhibit earlier onset and more severe symptoms compared to patients in previous case studies. Notably, proband II, who has a co-occurring *SCN5A* variant, shows a more severe arrhythmic phenotype, including frequent ventricular premature beats, intermittent and incomplete right bundle branch block, QT interval prolongation, atrial premature beats, and epsilon waves in leads V1-V3. The impact of these mutations can vary significantly due to genetic background, environmental factors, and the presence of other mutations. Therefore, more research and case studies are needed to further understand the phenotypes caused by compound mutations. Previous studies have reported that patients with the homozygous *DSG2* variant usually present with heart failure or arrhythmia attacks between 30–50 years old. Moreover, SCD is rarely observed in such patients. In this study, the onset ages of the three patients were significantly earlier than the cases reported in the previous studies and the clinical presentation was more severe. In 2019, a study by Chen et al. indicated that a single heterozygous variant in *DSG2* might not be sufficient to lead to the ARVC phenotype, only patients who had MYH7 p.T441M variant of distal myopathy or carried the *DSG2* homozygous variant would manifest early-onset severe symptoms of ARVC, which suggests that other regulatory genes or environmental factors may enhance the pathogenicity and penetrance of DSG2 p.F531C (Chen et al., 2020). A 2019 study mentioned that among eight *DSG2* c.1592T>G (p.F531C) homozygous variant carriers without additional variants, each patient was diagnosed with ARVC based on the revised 2010 Task Force Criteria. The age at which ARVC-related symptoms first appeared ranged from 14 to 34 years. In comparison, the three probands in our study, who have the homozygous DSG2 c.1592T>G (p.F531C) variant along with additional variants, exhibited earlier onset, more acute presentation, and more severe symptoms (Chen et al., 2019a). Additionally, many studies have shown that individuals with multiple variants typically have more severe conditions compared to those with a single variant (Xu et al., 2010), which is consistent with our research findings. In conclusion, these results suggested that patients with multiple pathogenic variants should be carefully monitored for symptoms associated with arrhythmias and heart failure. Moreover, these patients should be treated early with ICD implantation device or other available treatments (Bhonsale et al., 2015). Left ventricular involvement and biventricular failure are common in patients with the homozygous DSG2 p. F531C variant; moreover, left ventricular involvement occurs early in life, especially in patients with combined *DSG* and *DSP* variants (Pilichou et al., 2006; Syrris et al., 2006; Basso et al., 2011; Chen et al., 2019a). We detected combined *DSP* and *DSG* gene mutations in proband I associated with the left heart enlargement, as detected on echocardiography. This was similar to previous findings (Chen et al., 2019a). WES has emerged as a valid and favorable technique for the molecular diagnosis of ACM. More studies and cases have verified the importance of WES has been previously used to detect the underlying molecular pathogenetic mechanisms in children with unexplained morbidity. Therefore, the use of WES can help patients to receive early diagnosis and timely treatment, including limitation of physical activity, application of beta-blocking drugs, and eventual implantation of an ICD device. Moreover, increasing popularity of WES is coinciding with decreasing price and shorter operation time. Therefore, WES is a primary diagnostic tool for physicians to determine the underlying genetic mutations that cause inherited cardiovascular disease in children. In the future studies, the underlying molecular mechanism by which the *DSG2* gene variant causes ACM needs to be further explored and experimentally verified.

## 5 Conclusion

In conclusion, we report three rare cases of ACM that shared the same homozygous *DSG2* c.1592T>G (p.F531C) variant in combination with additional cardiomyopathy-associated heterozygous mutations in the *DSP* c.7883T>C, *SCN5a* c.3577C>T, or *MYH7* c.427C>T genes. SCD occurred in all the three cases. The additional genetic variants aggravated the clinical manifestations of ACM caused by the homozygous *DSG2* mutation. Moreover, our data showed that the complex genetic disorders induced early disease onset and lethal cardiac events. Therefore, our data suggested that comprehensive genetic evaluation was necessary to identify the potential additional variants that may significantly influence clinical prognosis and outcomes. This would help in designing personalized therapeutic strategies for such patients.

## Data Availability

The data presented in the study are deposited in the GSA database (ngdc.cncb.ac.cn/gsub/) repository, accession number HRA008278.
